# Spectroscopic characterization of bacterial colonies through UV hyperspectral imaging techniques

**DOI:** 10.3389/fchem.2025.1530955

**Published:** 2025-02-18

**Authors:** Josune J. Ezenarro, Mohammad Al Ktash, Nuria Vigues, Jordi Mas Gordi, Xavi Muñoz-Berbel, Marc Brecht

**Affiliations:** ^1^ Departament Genètica i Microbiologia, Universitat Autònoma de Barcelona, Barcelona, Spain; ^2^ Process Analysis and Technology PA & T, Reutlingen University, Reutlingen, Germany; ^3^ Institut de Microelectrònica de Barcelona (IMB-CNM, CSIC), Universitat Autònoma de Barcelona, Barcelona, Spain; ^4^ CIBER de Bioingeniería, Biomateriales y Nanomedicina, Instituto de Salud Carlos III, Madrid, Spain

**Keywords:** hyperspectral imaging, UV spectroscopy, principal component analysis, discriminant analysis, colony identification

## Abstract

**Introduction:**

Plate culturing and visual inspection are the gold standard methods for bacterial identification. Despite the growing attention on molecular biology techniques, colony identification using agar plates remains manual, interpretative, and heavily reliant on human experience, making it prone to errors. Advanced imaging techniques, like hyperspectral imaging, offer potential alternatives. However, the use of hyperspectral imaging in the VIS-NIR region has been hindered by sensitivity to various components and culture medium changes, leading to inaccurate results. The application of hyperspectral imaging in the ultraviolet (UV) region has not been explored, despite the presence of specific absorption and emission peaks in bacterial components.

**Methods:**

To address this gap, we developed a predictive model for bacterial colony detection and identification using UV hyperspectral imaging. The model utilizes hyperspectral images acquired in the UV wavelength range of 225–400 nm, processed with principal component analysis (PCA) and discriminant analysis (DA). The measurement setup includes a hyperspectral imager, a PC for automated data analysis, and a conveyor belt system to transport agar plates for automated analysis. Four bacterial species *(Escherichia coli, Staphylococcus, Pseudomonas, and Shewanella)* were cultured on two different media, Luria Bertani and Tryptic Soy, to train and validate the model.

**Results:**

The PCA-DA-based model demonstrated high accuracy (90%) in differentiating bacterial species based on the first three principal components, highlighting the potential of UV hyperspectral imaging for bacterial identification.

**Discussion:**

This study shows that UV hyperspectral imaging, coupled with advanced data analysis techniques, offers a robust and automated alternative to traditional methods for bacterial identification. The model's high accuracy emphasizes the untapped potential of UV hyperspectral imaging in microbiological analysis, reducing human error and improving reliability in bacterial species differentiation.

## 1 Introduction

Pathogenic bacteria are responsible for many healthcare and safety problems, including infectious diseases (He et al., 2023), food poisoning (Hussain, 2016), and water pollution (Some et al., 2021). Due to their infectivity and rapid proliferation, fast and accurate methods for bacterial detection and identification are necessary to reduce the time lapse for decision-making and, thus, minimize healthcare risks, ecosystem impact, and economic losses associated with microbial pathogens. Different methods already exist for pathogen detection and identification based on bacterial cell culture on agar plates (van Belkum and Dunne, 2013), immunological detection (e.g., enzyme-linked immunosorbent assay) (Wyatt et al., 1993), molecular biology techniques like the polymerase-chain reaction (Maurin, 2012; Yamamoto, 2002), DNA microarrays (Call et al., 2003), biosensors (Boehm et al., 2007; Ahmed et al., 2014), or the use of specific reagents sensitive, for example, to bacterial metabolism (Ghatole et al., 2020; Hsieh et al., 2018) or the presence of Adenosine triphosphate (ATP) (Liu et al., 2023), among others (Chen et al., 2018; Dietvorst et al., 2020). However, the traditional plate culturing method is still the gold standard in pathogen detection and identification due to its simplicity, low cost, robustness, and reliability (Rohde et al., 2017), being the one included in the regulations for bacterial pollution assessment (Word Health Organization, 2017).

In practice, plate culturing involves bacterial growth in agar plates until the formation of monoclonal colonies is visually observable. Colonies from different bacterial species differ in morphology, color, shine, and opacity, among others, being distinguishable by experts after careful observation, sometimes under the microscope. Thus, plate culturing is, to some extent, susceptible to human errors. Apart from that, the technique’s main limitation is its long duration. Typically, bacterial proliferation until colony formation takes more than 18 h, being necessary more than 3–4 days in the case of slow proliferating bacteria (Franco-Duarte et al., 2023; Rajapaksha et al., 2019; Lee et al., 2020). One extreme situation is *Legionella*, which requires non-standard treatments and a second plate culturing for a proper diagnosis, thus delaying bacterial identification up to some weeks (Tronel and Hartemann, 2009; McDade, 2009).

One possibility to reduce the measurement time and speed up decision-making is to implement advanced imaging systems that are able to detect the colonies and identify them at an early stage of formation (Wang et al., 2020). In this sense, hyperspectral imaging is advantageous since it provides high-resolution images in a 3D data matrix or hypercube format, where two dimensions correspond to the spatial information (x, y coordinate) and the third one to spectroscopic data from each individual pixel (λ coordinate) (Gowen et al., 2015; Arrigoni et al., 2017). This large amount of information is generally processed using chemometrics to identify patterns in the data sets, which are not noticeable with the bare eye, and to create predictive models able to classify new data (Huang, 2022). Principal component analysis (PCA) has been commonly used in combination with hyperspectral imaging to reduce the spectral image data sets into representative variables called principal components (PCs) (Abdi and Williams, 2010). These PCs can then be used to perform PCA-based discriminant analysis (PCA-DA) (Uddin et al., 2021) with statistical techniques like quadratic discriminant analysis (QDA), which is used for data classification. Once selected and trained with samples of known nature and composition, the algorithm determines the probability of a new data point belonging to each class based on its features and assigns it to the class with the highest probability (Ghosh et al., 2021).

The combination of hyperspectral imaging and data analytics has already been used for microbial identification since each bacterium presents a unique pattern of absorption/emission, which can be utilized as a distinctive identifier or fingerprint. Hence, Turra et al. (2015) demonstrated the capability of VIS-NIR hyperspectral imaging in the wavelength range between 400 and 1,000 nm to distinguish the colonies corresponding to five pathogenic bacteria commonly related to urinary tract infections (UTI) (Turra et al., 2015). As a step forward, Guillemont et al. (2016) reduced the visible–near infrared (VIS-NIR) spectral resolution to 14 channels, each one corresponding to a single wavelength, to classify UTI-related bacterial colonies grown on chromogenic agar media (Guillemot et al., 2016). On the other hand, Kammies et al. (2016) employed NIR hyperspectral imaging between 900 and 2,500 nm to differentiate between Gram-positive and Gram-negative bacterial colonies (Kammies et al., 2016). Additionally, Gu et al. (2020) investigated the capacity of hyperspectral imaging to classify bacterial colonies regardless of the agar media used for their cultivation (Gu et al., 2020). Hence, these hyperspectral imaging studies for bacterial colonies identification on agar plates only considered the VIS-NIR regions, while UV radiation was not explored, even when many bacterial molecular components, including amino acids, pigments, and proteins, e.g., cytochromes (Ghosh et al., 2015; Orlandi et al., 2022; Gao and Garcia-Pichel, 2011), absorb in this region.

Here, UV hyperspectral imaging is used to develop a predictive chemometric model based on PCA-DA to distinguish colonies from bacterial species commonly present in water or clinical samples. Four different bacterial strains commonly related to clinics, food processing, or water distribution were used as model microorganisms to create and validate the predictive model. *Staphylococcus* and *Pseudomonas* were selected due to their clinical relevance since they are common pathogens implicated in nosocomial clinical infections (Hassan et al., 2021). *Escherichia coli* is one of the most common pathogens in water, commonly employed as a standard fecal contamination indicator. It has also been related to gastrointestinal tract, urinary tract, bloodstream, and central nervous system-related disease (Word Health Organization, 2017; Croxen et al., 2013; Mead and Griffin, 1998; Ishii and Sadowsky, 2008). Finally, some *Shewanella* species have been postulated as opportunistic pathogens. This microorganism can be found in either water or soil samples, although also isolated from food (milk, butter, eggs, poultry, raw fish, and beef products) (Vignier et al., 2013; Gruyter and Paździor, 2016; Ng et al., 2022). Additionally, this microorganism produces shiny and reddish/brown color colonies due to the presence of hemo groups in their large variety of cytochromes c (Gruyter and Paździor, 2016), which makes it a very attractive candidate for UV-hyperspectral detection (Mead and Griffin, 1998).

## 2 Materials and methods

### 2.1 Bacterial agar plate sample preparation

In this study, four different bacterial strains were cultivated: *Escherichia coli* (ATTC 10536), *Pseudomonas putida* (DMSZ 291), *Staphylococcus arlettae* (CVD059), *Shewanella oneidensis* (ATCC 700550). All bacteria were cultivated in Luria Bertani (LB) broth except *Shewanella oneidensis*, cultivated in Tryptic Soy (TS) media.

Cell suspensions were grown overnight (18 h) in the corresponding liquid culture media to prepare agar plate samples for UV hyperspectral imaging. The optical density of the overnight culture was measured and diluted to a cell concentration of approximately 10^8^ CFU·mL^-1^ (OD 0.12). Afterward, four individual spots were created for each bacterial culture plate, inoculating 5 µL of cell suspension per spot. Plates corresponding to *Escherichia coli* (EC) and *Staphylococcus* *arlettae* (SA) were incubated at 37°C, while *Pseudomonas putida* (PP) and *Shewanella oneidensis* (SO) at 30°C.

### 2.2 Set-up for UV-Hyperspectral imaging

**FIGURE 1:**
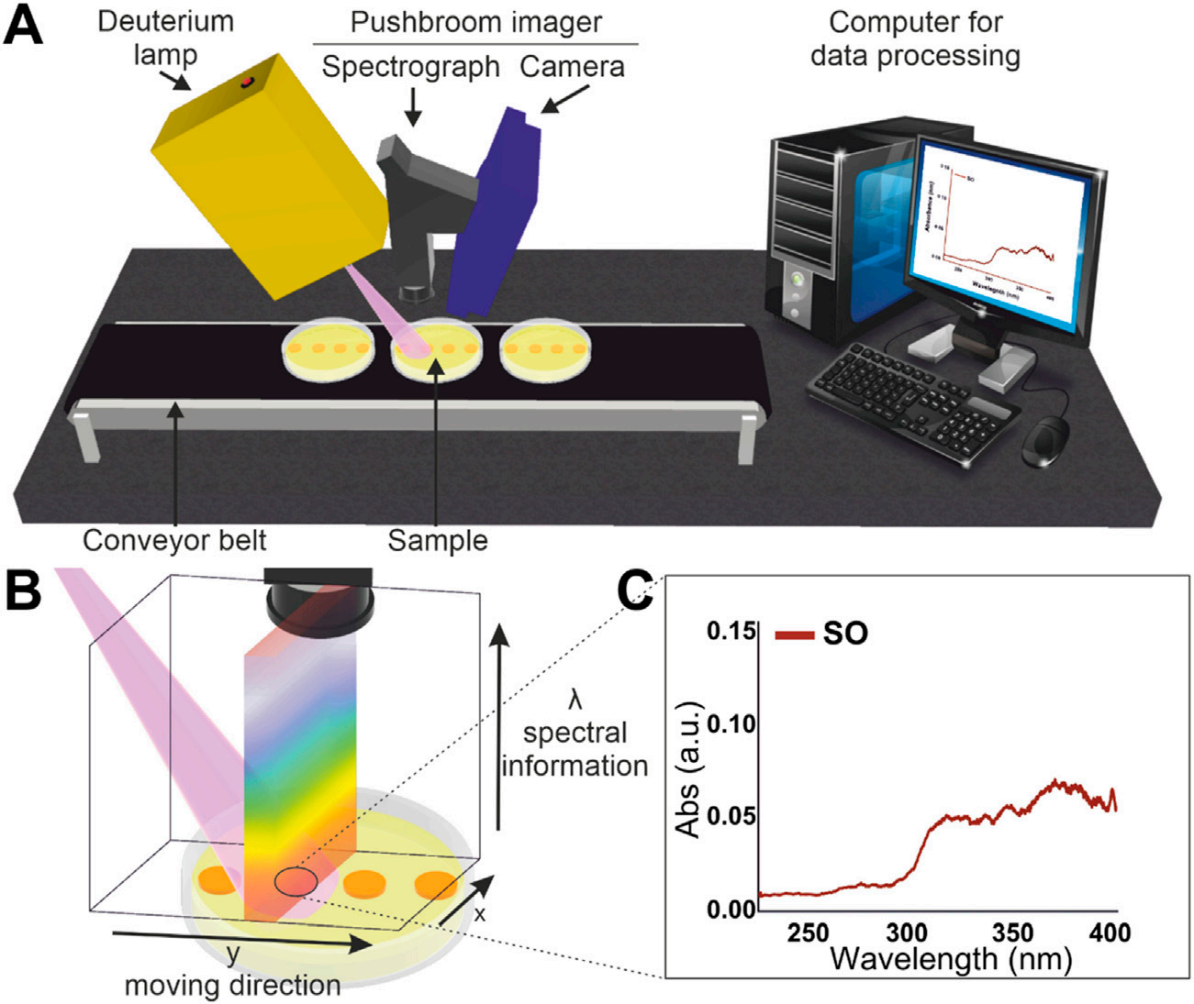
**(A)** Set-up for push-broom UV-hyperspectral imaging. **(B)** Representation of the push-broom image scanning principle. **(C)** Average UV spectrum extracted from pixels of a particular area of interest.

The set-up used for UV hyperspectral imaging was adapted from the system described in a previous work (Al Ktash et al., 2023). Figure 1 illustrates the UV hyperspectral imaging system based on a back-illuminated CCD camera (Apogee Alta F47: Compact, inno-spec GmbH, Nürnberg, Germany) and a spectrograph (RS 50–1938, inno-spec GmbH, Nürnberg, Germany) with a slit width of 30 μm. The CCD camera has a resolution of 1,024 × 1,024 pixels (spatial × spectral) and a pixel size of 13 μm × 13 μm. The integration time was optimized and set at 300 m to measure bacterial colonies. The set-up also incorporates a Deuterium lamp (SL3 Deuterium Lamp, StellarNet Inc., Tampa, Florida) as the light source. The hyperspectral set-up contains a conveyor belt (700 mm × 215 mm × 60 mm, Dobot Magician, Shenzhen Yue-jiang Technology Co., Ltd., Shenzhen, China), where samples are placed and displaced until the detection area. The conveyor belt moves at a constant speed of 0.15 mm/s.

### 2.3 Data collection and preprocessing

The UV hyperspectral imaging data were acquired with the SI-Cap-GB version V3.3.x.0 software (inno-spec GmbH, Nürnberg, Germany), which automatically calculated the values of the reflectance measurements. In order to correct the illumination variations during the scanning line and the scattering produced by the different topographies of the bacterial colonies, raw spectra corresponding to each pixel were converted to reflectance spectra after radiometric calibration using the following Equation 1 (Boldrini et al., 2012).

$$Reflectance=\frac{R}{R_{0}}=\frac{I_{sample-}I_{dark}}{I_{reference-}I_{dark}}$$ (1)

where R is the intensity reflected from the sample, and R_0_ is the intensity reflected from a specific reference material with high reflectivity, in this case, Polytetrafluoroethylene (PTFE) or Teflon). I_sample_ corresponds to the spectral intensity of the sample, I_dark_ is the intensity of the dark, and I_reference_ is the intensity of the reference sample, in this case PTFE. Absorption is calculated as the negative logarithm of reflectance (Abs = -log (R/R_0_)). All the data presented in the graphs of this research article are represented using absorbance, calculated as the logarithm of the reflectance.

The acquired hyperspectral data were analyzed using the HydraPCA software, a custom-made program in Matlab (MATLAB 9.2.0, Mathworks, MA, United states), which was designed to extract spectra from individual pixels. Considering the sensor size (1,024 pixels × 1,024 pixels), the program analyzed 1,024 pixels in the x-direction (lateral resolution in x), and 1,024 pixels in the wavelength λ direction ranging from 225 nm to 410 nm (variables/columns in the PCA matrix). By moving the conveyor belt line by line, a lateral resolution in y is realized.

### 2.4 Multivariate data analysis and model building

Multivariate data analysis was carried out with Aspen Unscrambler™ version 10.5.1″(Aspen Technology Inc., Bedford, MA, United states). First, UV spectra were preprocessed to calculate PCA. Linear baseline correction and Savitzky–Golay smoothing (5 points, symmetric, 3rd polynomial order) were carried out. PCA models were then calculated with mean centering, cross-validation, and the NIPALS algorithm. QDA was used with mean centering and segmented cross-validation for model building according to the sample type and the Kernel algorithm.

The prediction performance was analyzed by calculating the accuracy (Equation 2), specificity (Equation 3), and sensitivity (Equation 4) of the model (Dankers et al., 2019).

$$Accuracy=\frac{\left(TN+TP\right)}{\left(TN+TP+FP+FN\right)}$$ (2)

$$Specifity=\frac{TN}{\left(TN+FP\right)}$$ (3)

$$Sensitivity=\frac{TP}{\left(TP+FN\right)}$$ (4)

where TP is true positive, FP is false positive, TN is true negative, and FN is false negative prediction.

## 3 Results

### 3.1 UV absorption spectra of bacterial agar plate colonies

The four bacterial species selected for this research—*Escherichia coli, Pseudomonas, Staphylococcus, and Shewanella*—exhibit distinct characteristics while sharing traits that make them suitable for UV-based detection (details in Table 2). Both *Escherichia coli* and *Pseudomonas* are gram-negative bacteria. *E. coli* is a significant pathogen in the healthcare field and a widely used model organism in research, while *Pseudomonas* is notable for its environmental importance and its role as an opportunistic pathogen, particularly in immunocompromised individuals. *Pseudomonas,* produces pigments like pyocyanin which offer unique spectral features under UV light, whereas *E. coli* lacks pigments but absorbs UV light through its structural components, such as proteins and nucleic acids. *Staphylococcus*, a gram-positive bacterium, is medically significant due to its involvement in various infections and produces pigments like staphyloxanthin. *Shewanella*, another gram-negative bacterium, has also been reported as an opportunistic pathogen which contains a wide variety of light absorbing c-cytochromes. Despite differences in morphology, pigmentation, and ecological roles, all four species demonstrate potential for differentiation using UV hyperspectral imaging.

Bacterial agar plate colonies were produced for the four bacterial strains [*Escherichia coli* (ATTC 10536), *Pseudomonas putida* (DMSZ 291), *Staphylococcus arlettae* (CVD059), *Shewanella oneidensis* (ATCC 700550)]. The four colonies were grown until reaching a minimum size of 13 µm in diameter, corresponding to the hyperspectral imager resolution (13 μm × 13 µm). Then, the plates were analyzed using UV hyperspectral imaging in the wavelength range between 225 and 400 nm. Control agar plates with LB and TSA without bacteria were also prepared and used as control samples.

**FIGURE 2:**
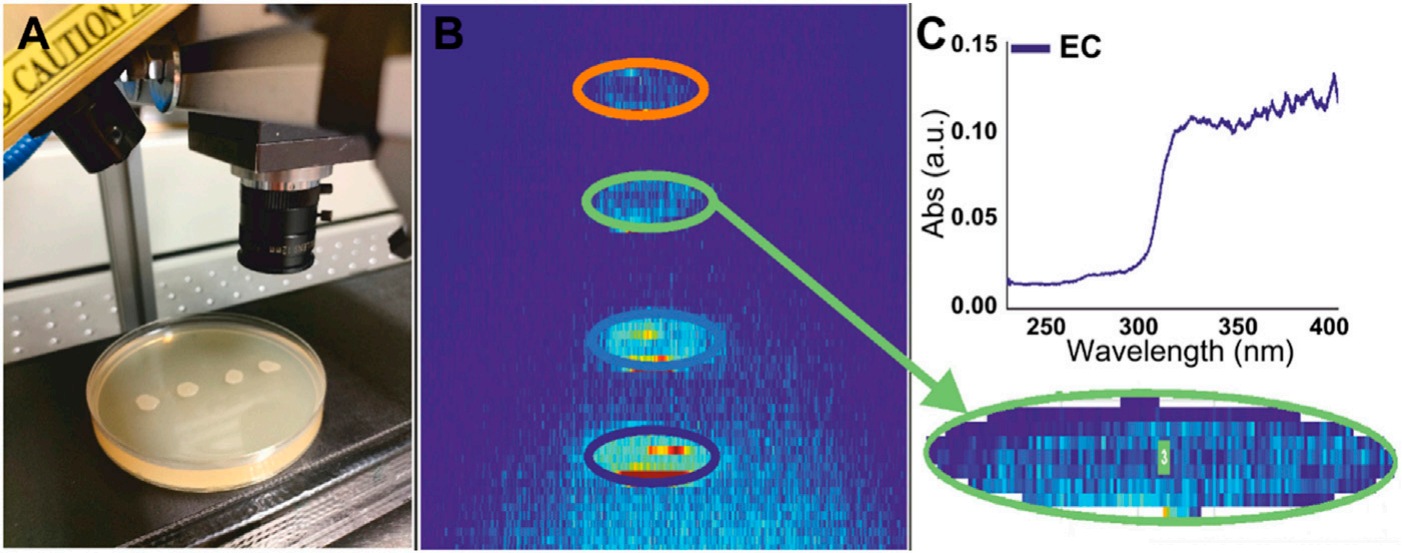
**(A)** Photo of a LB agar plate with 4 *Escherichia coli* spots placed on the belt under the UV lamp, with the hyperspectral camera ready for measurement. **(B)** UV-hyperspectral image taken from the sample. Regions of interest corresponding to the four *Escherichia coli* spots are indicated with different color circles. **(C)** Average absorption spectra extracted from one of the selected regions of interest.

Figure 2 shows images of the different steps carried out for the UV-hyperspectral image analysis of the colonies on agar plates. First, agar plates under analysis were located on the conveyor belt. Once reaching the detection area (i.e., below the lamp, Figure 2A), the camera took hyperspectral images in reflectance mode (Figure 2B). As shown in Figure 2B, the bacterial colonies could be distinguished from the surrounding medium, being able to count the number of colonies in the sample, i.e., in this case four. The area corresponding to each colony contained multiple pixels (between 220 and 250 pixels in the case of overnight cultures of *Escherichia coli*), which could be easily selected for bacterial identification through spectral analysis. Even when each pixel provided an independent UV-spectrum, the average spectrum of the 220–250 individual pixels was considered representative of the specific bacterial colony and used to compare colonies between them (Figure 2C).

**FIGURE 3:**
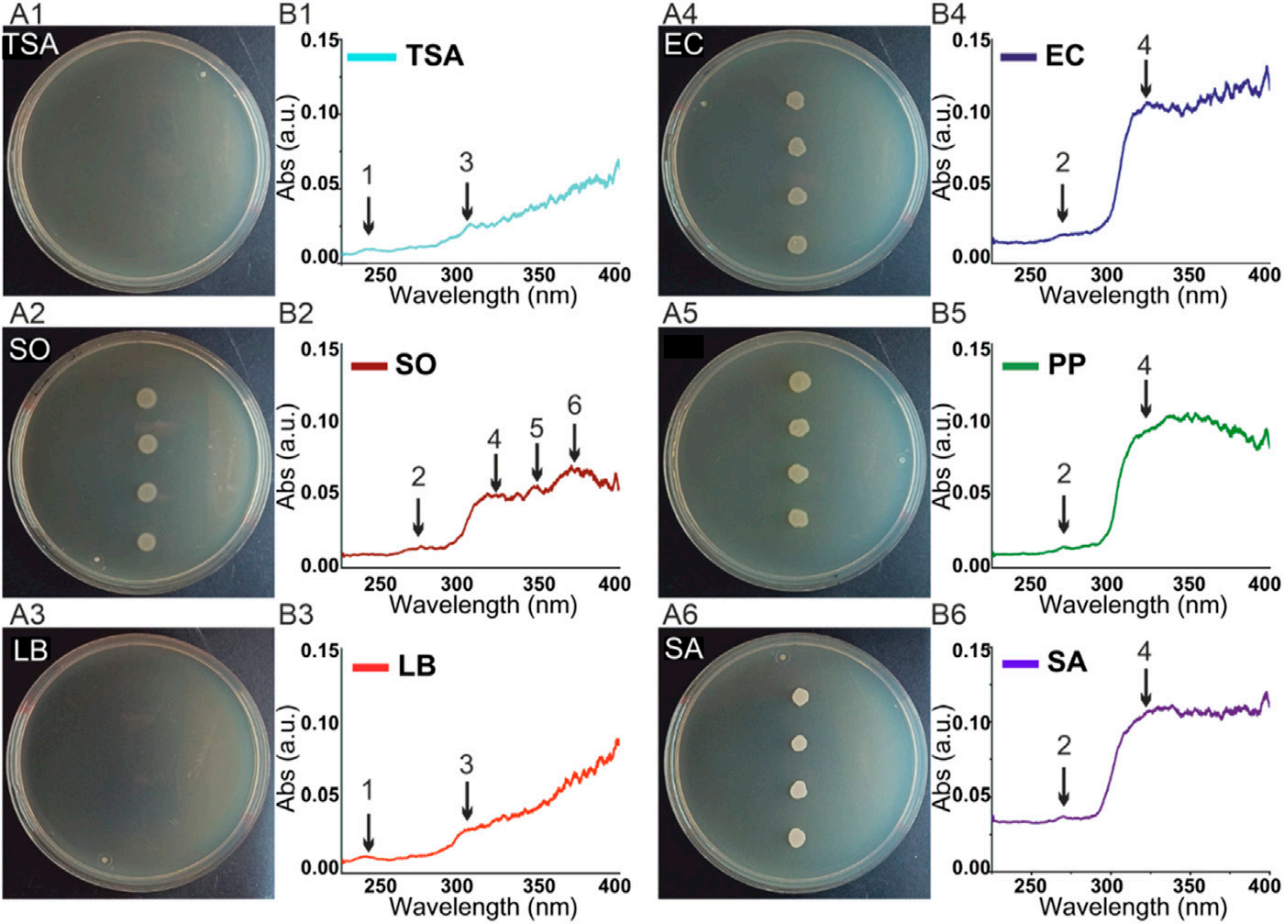
**(A)** Photo of agar plates **(B)** Average absorption spectra of the analyzed samples. Numbers correspond to the following sample: 1) Tryptic soy agar (TSA) control; 2) *Shewanella oneidensis* (SO); 3) Luria Bertani (LB) agar control; 4) E*scherichia coli* (EC); 5) *Pseudomonas Putida* (PP); 6) *Staphylococcus arlettae* (SA).

Representative agar plates of each sample type are presented in Figures 3A1–6. In addition to an agar plate containing four colonies for each bacterial strain (*Shewanella oneidensis*, *Escherichia coli*, *Pseudomonas putida* and *Staphylococcus arlettae*), a control agar plate was analyzed for each agar type (LB and TSA)—six plates in total. As shown, the colonies corresponding to each bacterial strain differed in morphology, topography, and color. This resulted in species-specific absorbance spectra for each bacterial type, i.e., bacterial fingerprints, which should permit the differentiation and identification of each bacterial strain (Figure 3B). It can be observed that the spectra from LB and TSA agar (Figures 3B1, 3B3) clearly differed from plates containing bacterial colonies. LB and TSA agar presented a small peak near 230 nm (peak 1), which was not observed in bacterial colonies. In contrast, the samples containing bacteria colonies show peaks between 260 and 280 nm (Figures 3B2–B6; peak 2) and a shoulder in the range between 290 nm and 320 nm (Figures 3B2–B6; peak 4). Variances from 300 nm to higher wavelengths are probably related to the different biochemical composition of bacteria, e.g., the presence of specific pigments.

### 3.2 Principal component analysis and model building

In each agar plate, four discrete colonies were grown and used to acquire hyperspectral images. Three of the colonies were used to create a model and the fourth one was employed for future sample prediction.

**FIGURE 4:**
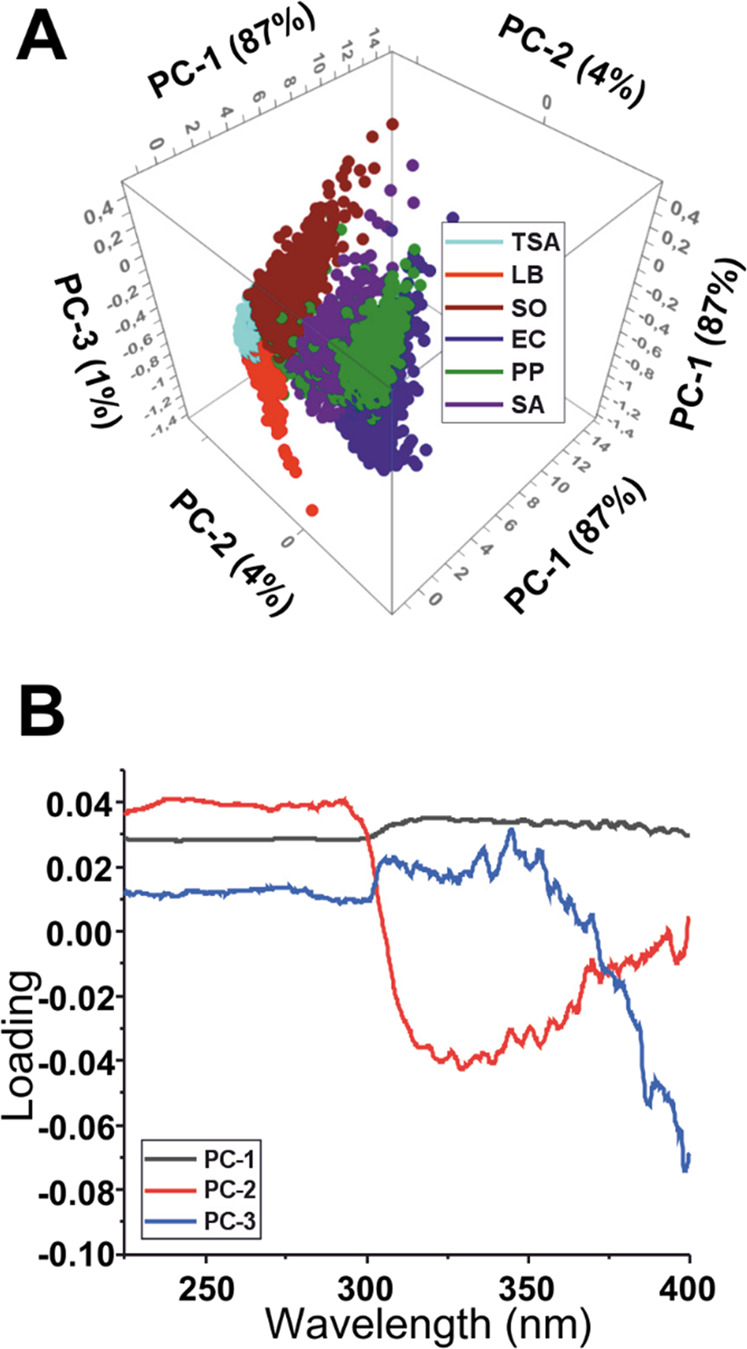
**(A)** 3D score plot of the PCA analysis results for PC1, PC2, and PC3. **(B)** Loadings plot of the three PCs.

Principal component analysis (PCA) was used to extract the essential spectral characteristics of all spectroscopic data obtained from hyperspectral imaging (Uddin et al., 2021), thus enabling straightforward data analysis and visualization. The 3D score plot of the PCA model created for the agar and bacterial colony samples at each sample pixel is shown in Figure 4A. This model explains 92% of the total variance of the spectral changes by the first three principal components. The first principal component PC1 (87%), captures the largest amount of data. The following components, PC2 (4%) and PC3 (1%), capture the remaining variation in descending order. The closer the samples are in the score plot, the more similar they are concerning the three components.

The PC1 variable influences 87% of the recorded data and, as indicated by the loadings, it has a constant positive correlation along the spectra without significant variations. On the other hand, PC2 and PC3 have more variations and, as can be observed in the 2D score plot for components PC2 and PC3 (Supplementary Figure S1), they are responsible for a better grouping of the different samples. All bacteria except SO have a negative correlation with PC2 and differ in the correlation to PC3, which simplifies their classification. The difference in PC2 will make SO better identified against the other bacterial species. On the other hand, when considering the samples that are positive in PC2, LB agar is negatively correlated to PC3, TSA has a close to null relation, and SO is positively related. Hence, the samples can be distinguished and grouped only considering three PCs. The loading plot (Figure 4B) also indicates that the most significant variation in the PCs starts around the 290 nm wavelength. Thus, in future experiments, the analyzed spectral range may be reduced, making it possible to decrease the amount of collected data.

In order to validate the quality of the PCA model, the scores of the first three PCs were used to make a quadratic discriminant analysis (PCA-QDA). For each sample, PCA-QDA gives a value that correlates with a group, and thus it is possible to determine the group to which the sample belongs to. The sample is assigned then to the corresponding group, either a bacterial species or an agar type. The model performance was tested with segmented cross-validation according to sample type (Avram et al., 2020).

**FIGURE 5:**
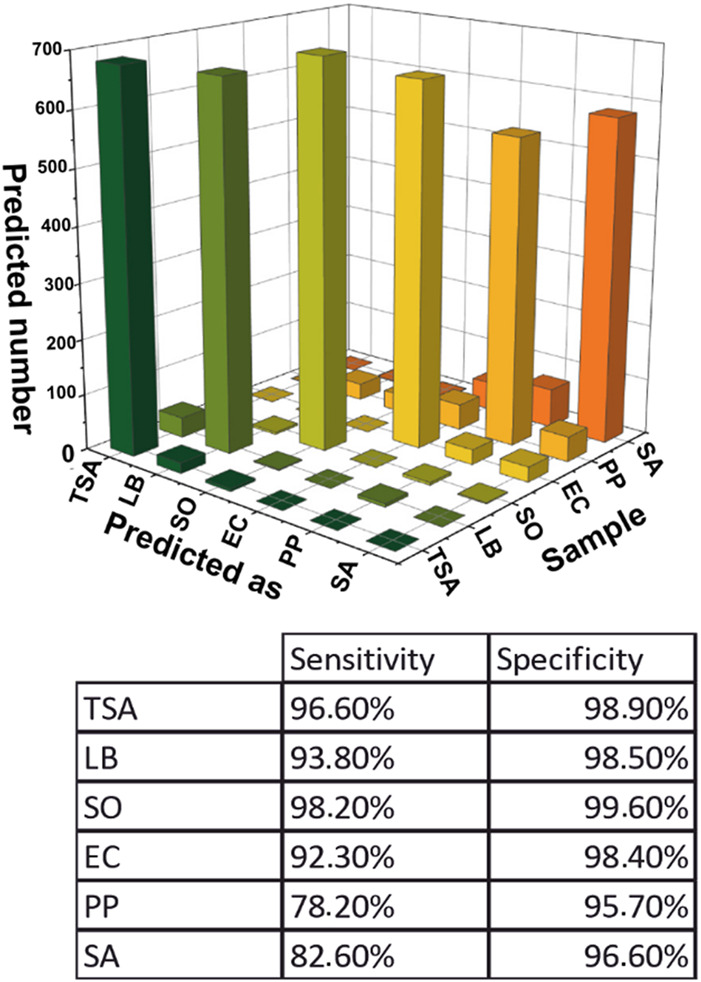
Graphical representation of the confusion matrix of PCA-DA model and the sensitivity and selectivity values for each sample.

Figure 5 illustrates the confusion matrix (Supplementary Table S1) for the agar media and bacterial colony samples, which evaluates the reliability of the classification model. It summarizes the number of correct and incorrect predictions made by the model on a data set by comparing the predicted and actual labels. The overall accuracy of the model was 90.3%. The high diagonal bars represent the accordance between the predicted and actual values, corresponding to dark-grey highlighted diagonal elements in Supplementary Table S1. The correctly predicted pixel number was much more significant than the incorrect predictions, corresponding to off-diagonal elements light-highlighted in Supplementary Table S1.

The sensitivity and specificity of the model were determined for all samples. Spectra corresponding to the TSA and LB agar were predicted with 96.6% and 93.8% of sensitivity and 98.9% and 98.5% of specificity. Most of the mispredicted ones corresponded to the other agar types. So, it could be concluded that the model can distinguish bacterial colonies from the agar. Regarding the bacterial colonies, SO was the best-predicted bacteria with 98.2% sensitivity and 99.6% specificity. The bacteria identified with the lowest sensitivity (78.2%) and specificity (95.7%) was PP.

Once the model was developed, it was used to predict the four colonies and certify its ability to identify different bacterial colonies. After analyzing the pixels corresponding to each colony, the probability of belonging to one of the four bacterial strains or the two agar types was determined with the current model (Table 1). The model indicated that there is a 98% chance for colony 1 to correspond to SO, 92% for colony 2 to be EC, 78% for colony 3 to be PP, and 83% for colony 4 to pertain to SA. Thus, all colonies could be correctly predicted after classifying them according to the highest probability.

**TABLE 1:** Prediction of the blind colony samples according to the classification of the spectra recorded from each single pixel forming their image. P (%) indicates the probability of pertaining to bacterial species.

	Colony 1	P (%)	Colony 2	P (%)	Colony 3	P (%)	Colony 4	P (%)
TSA	2	0.3	0	0	1	0.12	0	0
LB	3	0.43	0	0	31	4.4	1	0.15
SO	693	98.3	0	0	32	4.5	1	0.15
EC	0	0	650	92	45	6.4	49	7
PP	5	0.71	28	4	551	78	68	9.7
SA	2	0.3	27	4	45	6.4	582	83
Prediction	SO		EC		PP		SA	

## 4 Discussion

### 4.1 UV absorption spectra of bacterial agar plate colonies

UV-Hyperspectral images of colonies containing agar plates allowed for easy detection and selection of the areas of interest for the spectral analysis. One of the first differences to highlight was the peak at 230 nm, which was only present in the LB and TSA agar controls. This peak has been related to unfolded (denaturalized) proteins (Liu et al., 2009). The process of preparing the agar culture plates includes a sterilization step of 20 min at 121°C of the medium prior to plating. This high-temperature step denaturalizes the proteins present in the medium, being responsible for the appearance of this peak. In contrast, bacteria are seeded and grown at physiological conditions, so proteins and nucleotides associated with them and present on the surface of the plate after cell culturing are not denaturalized. This aspect was confirmed by the presence of absorption peaks between 260 and 280 nm (peak 2), which are associated with non-denaturalized proteins and nucleotides.

Spectral differences above 300 nm may be attributed to variations in the biochemical composition of bacteria, concretely to the presence of natural pigments, like carotenoids, flavins, phenazines, quinones, monascines, violaceins, indigoidines or melanins, able to absorb in the UV-VIS range (Celedón and Díaz, 2021). *Pseudomonas* species, for example, contain pyocyanin, pyochelin, pyoverdine, and pyomelanine (Srivastava et al., 2022; Hoegy et al., 2014; Hamad et al., 2020; Mould et al., 2020), *Staphylococcus* produce staphyloxantin and zeaxanthin (Pelz et al., 2005; Ram et al., 2020), and *Shewallena*, characterized to contain high concentration of a wide variety of c-cytochromes as part of its electron transport chain (Kaila and Wikström, 2021; Gennis, 1987), also presents pyomelanine as electron acceptor in the iron reduction process (Kuttan et al., 2023) (Table 2).

**TABLE 2:** Pigments or components found in *Pseudomonas spp*., *Staphylococcus spp*., *Shewanella* spp., *E. coli* and their absorption maxims in the UV-VIS range.

Pigment/component	Absorption peaks	Bacteria	Ref.
Pyocyanin	255.5, 306, 525, 691 (standard in chloroform)	*Pseudomonas* spp.	Hamad et al. (2020)
	225, 247, 284, 388, 555 (standard in HCl		
Pyoverdine	380 (pH 5) or 400 (pH 8)	*Pseudomonas* spp.	Hoegy et al. (2014)
Pyochelin	310	*Pseudomonas* spp.	Mould et al. (2020)
Pyomelanine	250–280	*Pseudomonas* spp.	Bayram (2021)
		*Shewanella*	
Staphyloxantin	463, 490	*Staphylococcus* spp.	Pelz et al. (2005)
Zeaxanthin	445, 472 (in dichloromethane)	*Staphylococcus* spp.	Murillo et al. (2019)
	451 (in ethanol)		
C-cytochrome	550	*All bacteria*	Gennis (1987)

### 4.2 Principal component analysis and colony identification model

The multivariate data analysis used to identify patterns and relationships between the spectra extracted from the samples showed that 92% of the total variance can be explained by using three PCs. PC1 has a constant positive correlation along the spectra without significant variations. On the other hand, PC2 and PC3 are responsible for the sample grouping, as evidenced in the 2D score plot (Supplementary Figure S1).

The model for bacterial colony identification by discriminant analysis generated based on the principal components showed an overall accuracy of 90.26%. Moreover, sensitivity and selectivity values for each bacterial strain and agar type were high and confirmed by the validation test made with the colonies used for prediction. These results suggest UV-hyperspectral can effectively discriminate between bacterial colonies based on their unique spectral signatures.

As can be observed in Table 3, previous approaches using hyperspectral imaging for colony identification in agar plates have been realized in the VIS-NIR spectral range. To the best of our knowledge, this study is the first to use UV-hyperspectral imaging for purposes and reach similar accuracy yields. Moreover, in this research, the agars used were similar, and for generic growth, there were no chromogenic and specific agars as in some of the research. It is well known that these types of agars generate changes in the appearance of some bacterial colonies, affecting their spectral properties and making their identification easier. However, they are more expensive than generic growth agars. The model developed in this research was not only able to detect colony growth in both culture media but also was able to distinguish between them, indicating that, if necessary, prediction models could be developed depending on the agar used for the growth.

**TABLE 3:** Comparative table of studies employing hyperspectral imaging for bacterial colony identification in agar plates.

Bacteria	Agar type	Wavelength (nm)	Model	Acc (%)	Ref
UTI pathogens *Escherichia coli* *Enterococcus faecalis* *Staphylococcus aureus* *Proteus mirabilis Proteus vulgaris Candida*	Chromogenic agar and Blood agar	VIS-NIR (400–1,000)	1) PCA + SVM 2) ROBPCA + RSIMCA	95	Turra et al. (2015)
UTI pathogens *E.coli, Proteus, Enterococci, Klebsiella, Enterobacter, Serratia, Citrobacter*	Chromogenic agar	VIS-NIR (400–1,000)	Lasso method	90–100	Guillemot et al. (2016)
*Bacillus cereus, Escherichia coli, Salmonella enteritidis* *Staphylococcus aureus* and *Staphylococcus epidermidis*	Luria-Bertani (LB) agar	NIR (900–2,500)	PCA + PLS-DA	82.0–99.96 (except for *E.coli ans S. enteirtis*)	Kammies et al. (2016)
*Escherichia coli, Staphylococcus aureus* and *Salmonella*	Luria–Bertani agar (LB), plate count agar (PA) and Tryptic soy agar (TSA)	VIS-NIR (400–1,000)	PLS-DA and GOA-SVM	<80(PLS-DA) 99 (GOA-SVM)	Gu et al. (2020)
*Escherichia coli, Listeria monocyogenes, Listeria seeligeri, and Staphylococcus aureus*	Tryptic soy agar (TSA)	VIS-NIR (400–1,000)	IWO-SVM	97	Feng et al. (2018)
*Eschecirichia coli, Staphylococcus arlettae, Pseudomonas putida, and Shewanella oneidensis*	Luria-Bertani (LB) and Tryptic soy agar (TSA)	UV (225–400)	PCA-QDA	90	This study

These results demonstrate that UV hyperspectral imaging has great potential in bacteria identification, offering a reliable alternative to the methods based on human interpretation. However, future approaches should be centered on increasing the number of bacterial species used to build a prediction model and try to cover the wide variety of bacterial species that can be found at the different scenes where plate culturing is used for identification purposes (i.e., environmental, clinical, food and water analysis). Yeast and molds of interest could also be included, as well as different agar types. Additionally, recent computer science and programming advances could allow for the automation of all the identification and quantification processes (Yoon et al., 2015). This automation could enhance efficiency and ensure the scalability and reproducibility of results.

## 5 Conclusion

In this study, we have successfully presented a proof of concept foreseeing the combination of UV hyperspectral imaging and chemometric analysis as a powerful and promising technology for identifying and counting bacterial colonies. This first approach employed three bacterial species of high interest in food, water, and clinical sample analysis (*E. coli*, *Staphylococcus*, *Pseudomonas*) and one control bacteria (*Shewanella*) to build a prediction model based on PCA-DA. The PCA model was able to classify all these samples with the first three PCs with a 90.3% overall accuracy, and the correct prediction of four blind samples confirmed the high sensitivity and selectivity of the generated model. So, the results indicate the potential of this technique as an attractive alternative to human-based agar colony identification, providing automated, reliable, fast, and user-friendly machine diagnosis. While recognizing the necessity for further research with a broad range of bacterial strains and culture media types, the results suppose an advancement in the application of this technology within the field of microbiology.

## Data Availability

The raw data supporting the conclusions of this article will be made available by the authors, without undue reservation.
