# Supplementary material for: Spectroscopic characterization of bacterial colonies through UV hyperspectral imaging techniques
Source: Front Chem. 2025 Feb 18;13:1530955. doi: 10.3389/fchem.2025.1530955 (PMC11876133; doi:10.3389/fchem.2025.1530955)
Supplement: Supplementary file 1 [file DataSheet1.docx]

Supplementary Material

# Supplementary Data

**
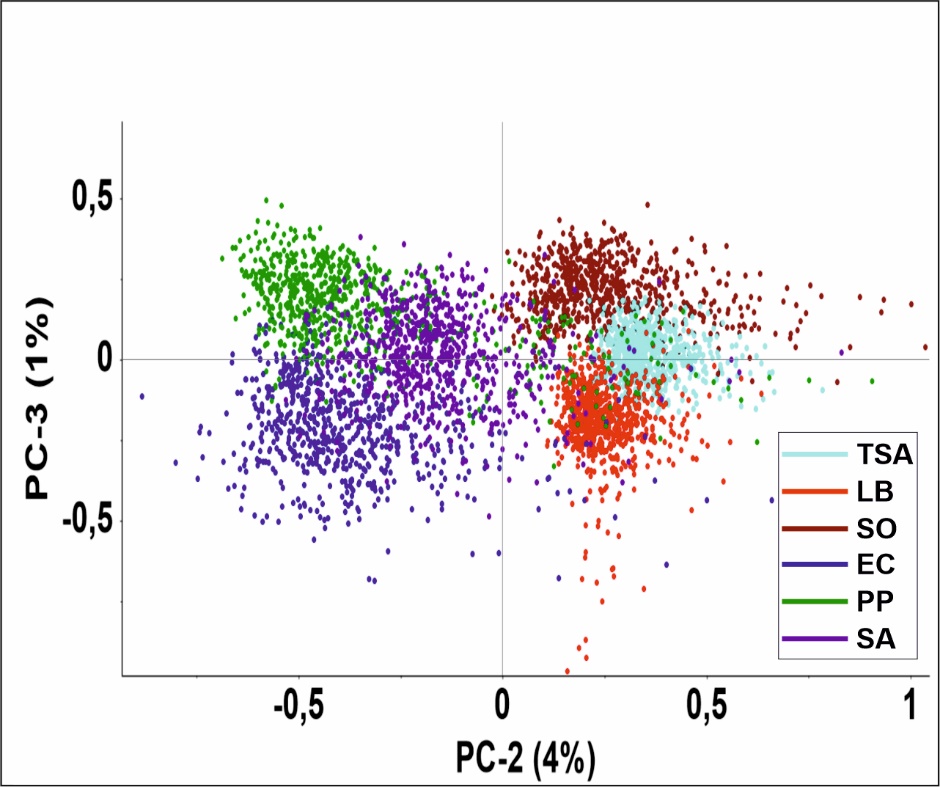
**

**Figure S1.** 2D PCA score plot for PC2 and PC3

**Table S1.** Model confusion matrix of PCA-DA model.

|  | TSA | LB | SO | EC | PP | SA |
| --- | --- | --- | --- | --- | --- | --- |
| TSA | 681 | 36 | 2 | 0 | 1 | 0 |
| LB | 19 | 661 | 4 | 0 | 30 | 1 |
| SO | 5 | 2 | 692 | 0 | 32 | 1 |
| EC | 0 | 0 | 0 | 651 | 46 | 51 |
| PP | 0 | 6 | 5 | 27 | 551 | 70 |
| SA | 0 | 0 | 2 | 27 | 45 | 582 |
